# ASSESSING THE RELATIONSHIP BETWEEN SERUM IGF-1 AND ADIPOSITY BY AGE IN THE LONG LIFE FAMILY STUDY

**DOI:** 10.1093/geroni/igz038.961

**Published:** 2019-11-08

**Authors:** Rehab A Sherlala, Candace M Kammerer, Allison L Kuipers, Mary K Wojczynski, Svetlana Ukraintseva, Mary Feitosa, Jonas Mengel-From, Ryan Minster

**Affiliations:** 1 University of Pittsburgh, Pittsburgh, Pennsylvania, United States; 2 Department of Human Genetics University of Pittsburgh; Pittsburgh, Pennsylvania, United States; 3 Department of Epidemiology University of Pittsburgh; Pittsburgh, Pennsylvania, United States; 4 Department of Genetics Washington University in St. Louis, Saint Louis, Missouri, United States; 5 Duke University, Durham, North Carolina, United States; 6 Washington University School of Medicine, St Louis, Missouri, United States; 7 University of Southern Denmark, Odense C, Denmark, Denmark

## Abstract

Serum levels of insulin-like growth factor 1 (IGF-1) and measures of adiposity, such as body mass index (BMI), are associated with susceptibility to age-related diseases. Previous reports of the relationship between IGF-1 and BMI ranged from positive to negative to no relationship, perhaps because previous reports studied different age cohorts. Using data on 4270 participants (aged 24-110 years) from the Long Life Family Study, we investigated the relationship between IGF-1 and BMI overall and by age groups. IGF-1 and BMI were positively correlated in the total sample (β=0.161, r2= 0.0038, p=1.8-05). However, further analyses revealed that the relationship between IGF-1 and BMI varied by age quartile: in the 1st quartile (24-58yo) the relationship was negative (β=−0.204, r2= 0.011, p=0.0008); in the 2nd quartile (59-66yo) the relationship was negative but non-significant (β=−0.069, r2= 0.0012, p=0.28); in the 3rd quartile (67-86yo) the relationship was positive but non-significant (β=0.106, r2= 0.002, p=0.13); and in the 4th quartile (87-110yo) the relationship was positive (β=0.388, r2= 0.019, p=1.2−05). This pattern did not differ by sex. We also detected a similar age-related pattern between IGF-1 and BMI using an independent dataset (NHANES III), comprising 2550 men and women aged 20-90 years. Our results may clarify some of the inconsistency in previous literature about the relationship between IGF-1 and BMI. Additional studies of IGF-1 and adiposity measures are needed to better understand the underlying mechanisms involved.

